# Artemisinin derivatives modulate KEAP1-NRF2-xCT pathway to alleviate Sjögren’s disease: insights from scRNA-seq and systems biology

**DOI:** 10.3389/fimmu.2025.1626230

**Published:** 2025-09-09

**Authors:** Yong Luo, Liuting Zeng, Yanan Wang, Qianyue Yang, Chang Liu, Xiaojun Tang, Genhong Yao, Lingyun Sun

**Affiliations:** ^1^ Department of Rheumatology and Immunology, Nanjing Drum Tower Hospital Clinical College of Nanjing University of Chinese Medicine, Nanjing, Jiangsu, China; ^2^ Department of Rheumatology and Immunology, Nanjing Drum Tower Hospital, Chinese Academy of Medical Sciences and Peking Union Medical College, Graduate School of Peking Union Medical College, Nanjing, China; ^3^ Department of Rheumatology and Immunology, Nanjing Drum Tower Hospital, Nanjing University Medical School, Nanjing, China; ^4^ Department of Rheumatology and Immunology, Nanjing Drum Tower Hospital, The Affiliated Hospital of Nanjing University Medical School, Nanjing, China

**Keywords:** scRNA-seq, systems biology, artesunate, Sjögren’s disease, ferroptosis

## Abstract

**Introduction:**

Sjögren’s Disease (SJD) is characterized by salivary gland dysfunction, and ferroptosis in salivary gland epithelial cells (SGECs) contributes to glandular damage. Artesunate (ART) exhibits therapeutic potential in inflammatory diseases, but its effect on SJD via regulating ferroptosis remains unclear.

**Methods:**

Female 8-week-old NOD/Ltj mice were randomized into model (saline) and ART groups (oral gavage). Daily water intake, weekly salivary flow rate, and body weight were monitored. After 8 weeks, spleen and submandibular gland indices were measured. scRNA-seq analyzed SJD patient profiles, and RNA-seq evaluated inflammatory pathway responses to ART. Submandibular glands were histologically examined via HE staining (lymphocytic infiltration scoring). Western blotting and immunofluorescence detected KEAP1, TFRC, xCT, NRF2, GPX4, IgG, and C3 expression in glands and SGECs; ROS and JC-1 levels in SGECs were also assessed. Molecular docking analyzed ART-KEAP1 affinity, and transmission electron microscopy evaluated mitochondrial morphology.

**Results:**

scRNA-seq and systems biology showed activated ferroptosis signaling post-ART. ART inhibited KEAP1-mediated ubiquitination/degradation of NRF2, upregulated xCT and GPX4, and downregulated TFRC in vitro and in vivo. This protected SGECs from ferroptosis, reducing glandular damage and preserving function in NOD/Ltj mice.

**Discussion:**

ART ameliorates SJD in NOD/Ltj mice by suppressing SGEC ferroptosis through the KEAP1-NRF2 pathway, highlighting its potential as a therapeutic agent for SJD.

## Graphical Abstract

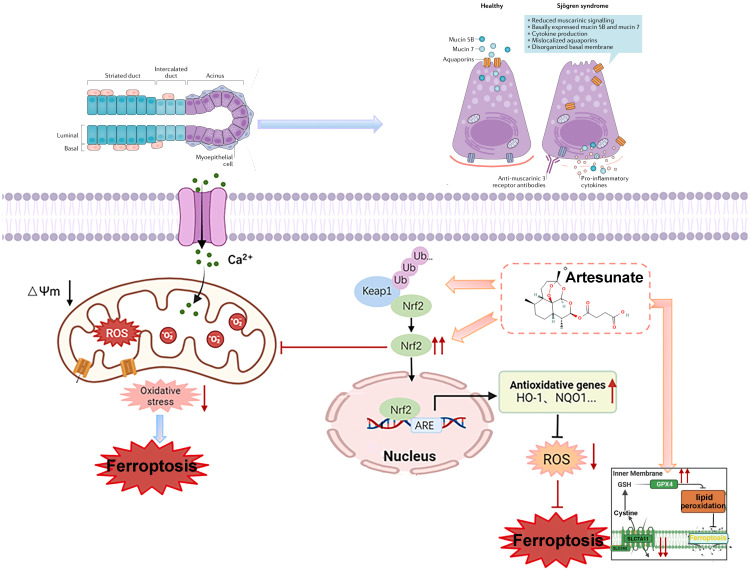

Graphical Abstract: Mechanism of artemisinin derivatives in alleviating Sjögren’s Disease via KEAP1-NRF2-xCT pathway modulation, inhibiting ferroptosis and restoring salivary gland function.

## Highlights

- Artesunate protects salivary gland cells from ferroptosis in Sjögren’s Disease mouse models.
- scRNA-seq and bulk RNA-seq analyses demonstrate that Artesunate modulates the KEAP1-NRF2-xCT pathway, effectively reducing oxidative stress and mitigating ferroptosis.
- Artesunate restores salivary gland function, offering a potential novel therapy for SJD.

## 1 Introduction

Sjögren’s Disease (SjD), a systemic autoimmune disorder predominantly affecting middle-aged to elderly women, is characterized by the dysfunction of exocrine glands, leading to hallmark symptoms such as xerostomia (dry mouth) and keratoconjunctivitis sicca (dry eyes) (1). Beyond these primary manifestations, SJD can progress to involve multiple organ systems, resulting in complications such as renal impairment, arthralgia, myalgia, and fatigue, all of which significantly impair patients’ quality of life (2). Despite advances in understanding its pathogenesis, current therapeutic strategies remain largely symptomatic, focusing on alleviating dryness symptoms rather than addressing the underlying mechanisms driving disease progression (3).This limitation underscores a critical unmet need for innovative treatments that target the root causes of glandular dysfunction in SJD.

While existing immunomodulatory therapies reduce exocrine gland inflammation, they fail to prevent SGEC death or restore glandular function, offering only suboptimal relief from symptoms.

Emerging evidence highlights the pivotal role of innate immune system activation in the early stages of primary Sjögren’s Disease (pSJD), with T cell activation—particularly Th17 cells—playing a central role in disease progression (4). Salivary gland epithelial cells (SGECs), which are essential for saliva production and maintaining oral hydration, are key targets of the inflammatory microenvironment in SJD (5). Chronic inflammation not only impairs SGEC function but also triggers their death, contributing to the severe secretory dysfunction observed in patients (6). While existing immunomodulatory therapies reduce exocrine gland inflammation, they fail to prevent SGEC death or restore glandular function, offering only suboptimal relief from symptoms (7, 8). This therapeutic gap highlights the urgent need to explore novel mechanisms underlying SGEC demise and identify effective interventions to preserve glandular integrity.

Recent research has identified ferroptosis—a form of regulated cell death driven by iron-dependent lipid peroxidation—as a critical contributor to SJD pathogenesis (9). Ferroptosis is marked by mitochondrial morphological changes, including contraction, loss of cristae, and cytoplasmic condensation, ultimately leading to cell membrane rupture and cell death (10, 11). In SGECs, this process is tightly regulated by antioxidant systems, including nuclear factor erythroid 2-related factor 2 (NRF2) and glutathione peroxidase 4 (GPX4) (12). Under physiological conditions, NRF2 regulates the expression of antioxidant genes, while GPX4 protects cells by converting harmful lipid hydroperoxides into harmless alcohols using glutathione (GSH) (13, 14). However, dysregulation of these pathways can render SGECs highly susceptible to ferroptosis, exacerbating glandular dysfunction (15) Additionally, increased cellular iron uptake via transferrin receptor 1 (TFRC) further enhances ferroptotic sensitivity, positioning TFRC as a potential biomarker for assessing ferroptosis in SJD (16, 17).

Despite growing recognition of ferroptosis in SJD, there remains a significant gap in understanding how this process can be therapeutically targeted to mitigate disease progression (18, 19). Artesunate (ART), a derivative of artemisinin widely used as an antimalarial agent, has emerged as a promising candidate due to its immunomodulatory properties. Studies suggest that ART modulates the balance of Th17/Treg cells in autoimmune disease models and inhibits macrophage migration by targeting macrophage migration inhibitory factor (MIF) (20, 21). Furthermore, ART inhibits STAT1 phosphorylation in endothelial cells, reducing MIF—a key regulator of SLE-associated atherosclerosis (22). Given that interferon-gamma (IFN-γ) suppresses the cystine-glutamate antiporter (system Xc-) via the JAK/STAT1 pathway—a critical step in inducing ferroptosis in SGECs—ART and its analogs may offer a novel therapeutic avenue for SJD (23, 24). However, the precise mechanisms by which ART modulates ferroptosis in SGECs and its potential efficacy in treating SJD remain poorly understood.

This study aims to address these gaps by investigating the therapeutic effects of ART on SJD using a Non-Obese Diabetic (NOD) mouse model (25). Specifically, we seek to determine whether ART ameliorates SJD symptoms by modulating ferroptosis in SGECs.

By addressing these questions, this study aims to provide mechanistic insights into the role of ferroptosis in SJD pathogenesis and establish ART as a potential therapeutic strategy. The findings could pave the way for the development of innovative treatments that not only alleviate symptoms but also target the underlying causes of glandular dysfunction, ultimately improving outcomes for patients with SJD.

## 2 Materials and methods

### 2.1 Animals and treatment

Non-Obese Diabetic (NOD/Ltj) mice and wild-type ICR mice were obtained from the Model Animal Research Center of Nanjing University. All animal experiments were approved by the Institutional Animal Care and Use Committee of Gulou Hospital, Medical School of Nanjing University (Approval No.: 2022AE01018). At 8 weeks of age, NOD mice were randomly divided into three groups: a vehicle-treated control group (n=10), and two treatment groups receiving artesunate (ART) at doses of 10.0 mg/kg (n=10) or 40.0 mg/kg (n=10). Age-matched and sex-matched inbred ICR mice served as normal controls (n=10).

Artesunate (MedChemExpress, Monmouth Junction, NJ, USA) was dissolved in a mixture of PEG300, Tween-80, and saline (stored at 4 °C). Mice in the ART-treated groups received daily intragastric administrations of ART at their respective doses for 8 weeks starting at 8 weeks of age. The vehicle-treated NOD/Ltj controls and normal ICR mice were administered the same volume of vehicle solution daily.

### 2.2 Saliva flow rate measurement

At 16 weeks of age, mice were anesthetized with a 1.4% sodium pentobarbital solution. Salivary secretion was stimulated by intraperitoneal injection of pilocarpine (3 mg/kg; MACKLIN, P875258). After 5 minutes, saliva was collected over a 15-minute period using pre-weighed 5 mL EP tubes containing dry cotton balls. Salivary flow rate (SFR) was calculated based on the weight difference between the initial and final weights of the tube and cotton ball, normalized to the mouse body weight, and expressed as mg/g/15 min (n=10) (19).

### 2.3 Histological examination

Salivary glands (SGs) were excised, fixed in 4% paraformaldehyde, and embedded in paraffin. Paraffin sections (5 µm thick) were stained with hematoxylin and eosin (H&E) for histological evaluation. Images were captured using an Olympus light microscope (Tokyo, Japan). Lymphocytic infiltration and fibrosis were quantified using ImageJ software (NIH, MD, USA) (n=10).

### 2.4 Single-cell RNA sequencing

Our analysis of scRNA-seq data published by Nayar et al. (2025) reveals that minor salivary gland single-cell RNA sequencing data from patients with Sjögren’s disease (SjD) were obtained from the GSE272409 dataset, which includes 7 patients with SjD and 6 patients with non-SjD sicca (26). The single-cell sequence data from the normal and disease groups were harmonized using the Harmony algorithm. Following quality control, a total of 94,000 single cells were sequenced. Cells were clustered unsupervised using t-distributed stochastic neighbor embedding (t-SNE) based on their gene transcription profiles. Differential expression analysis was conducted using algorithms to identify differentially expressed genes, and Fisher’s exact test was used to determine statistical significance. Marker genes for each cell population were identified and used to infer cell types. Cell clusters were then annotated based on significant differential expression of genes in the clusters and identified using the FindAllMarkers function in the Seurat package. For detailed cluster annotations, please refer to S3.

### 2.5 Bulk RNA sequencing

Total RNA was extracted from salivary glands using Trizol reagent (Thermo Fisher Scientific, MA, USA). RNA purity and integrity were assessed using a NanoDrop^®^ spectrophotometer (IMPLEN, CA, USA) and an Agilent 2100 RNA Nano 6000 Kit (Agilent Technologies, CA, USA), respectively. Sequencing libraries were prepared using the VAHTS Universal^®^ V6 RNA-seq Library Prep Kit (NR604 – 01/02) according to the manufacturer’s instructions. Indexed libraries were sequenced on the Illumina NovaSeq 6000 S4 platform, yielding paired-end reads of 150 base pairs. Differential gene expression was analyzed using DESeq2, selecting genes with a log2 fold change ≥ 0.5 or ≤ -0.5 and p-values < 0.05. Pathway analyses were performed using Gene Ontology (GO) and Gene Set Enrichment Analysis (GSEA). Sequencing data were uploaded to NCBI BioProject under the Submission ID PRJNA1050802. Detailed transcriptome results are provided in S1.

### 2.6 Cell culture

The human submandibular epidermal carcinoma-derived epithelial cell line A253 (Servicebio, STCC13301P) was cultured in RPMI 1640 medium supplemented with 10% fetal bovine serum (FBS) under standard conditions (37 °C, 5% CO2). Cells were seeded in 6-well plates, and when the cell density reached 80%, they were stimulated with IFN-γ (100 ng/mL) for 24 hours to establish an *in vitro* model (23, 24). After stimulation, cells were treated with artesunate (2.5 μM, 5 μM, or 10 μM) or Liproxstatin-1 (2 μM) for an additional 24 hours.

### 2.7 Transmission electron microscopy

A253 cells were treated with IFN-γ (100 ng/mL) for 24 hours followed by artesunate (2.5 μM, 5 μM, or 10 μM) or Liproxstatin-1 (2 μM) for 24 hours. Cells were fixed with 0.25% glutaraldehyde in 0.1 M sodium cacodylate buffer (pH 7.0) for 2 hours, post-fixed with OsO4 in 0.1 M phosphate buffer (pH 7.0), dehydrated in a graded ethanol series, and embedded in Embed 812 resin. Thin sections (70 – 90 nm) were stained with uranyl acetate and lead citrate and imaged using a transmission electron microscope (FEI, Hillsboro).

### 2.8 Reactive oxygen species detection

Intracellular ROS levels were measured using the fluorescent probe DCFH-DA (UElandy, R6033). After treatment, cells were washed three times with serum-free medium, and DCFH-DA was added at a final concentration of 10 μM. Cells were incubated at 37 °C for 20 minutes, washed three times to remove unincorporated dye, and imaged using a fluorescence microscope (IX83, Olympus, Tokyo, Japan). A total of 11 images from different positions were randomly selected and recorded across the 3 samples in each group (n=3).

### 2.9 JC - 1 assay for mitochondrial membrane potential

Mitochondrial membrane potential was assessed using the JC - 1 Mitochondrial Membrane Potential Kit (UElandy, J6004L). Cells were rinsed three times with PBS and incubated with 1× JC - 1 dye in serum-free medium at 37 °C for 30 minutes. Fluorescence emission shifts from red (590 nm) to green (529 nm) were measured using flow cytometry or confocal microscopy to evaluate mitochondrial depolarization. A total of 11 images from different positions were randomly selected and recorded across the 3 samples in each group (n=3).

### 2.10 Determination of glutathione (GSH) and malondialdehyde (MDA)

At the endpoint of the experiment, salivary gland lysate was collected. GSH and MDA were analyzed in lysates by the GSH detection kit (Beyotime, S0053) and MDA detection Kit (Beyotime, S0131), respectively (n=5).

### 2.11 Cell viability assay

Cell viability was assessed using the CCK - 8 assay (NCM Biotech, C6005). A253 cells were seeded in 96-well plates at a density of 4,000 cells per well. After treatment with IFN-γ (100 ng/mL) for 24 hours and subsequent treatment with artesunate (2.5 μM, 5 μM, or 10 μM) or Liproxstatin-1 (2 μM) for 24 hours, CCK - 8 reagent was added, and optical density was measured at 530 nm using a microplate reader (n=4).

### 2.12 Flow cytometry

Apoptosis was evaluated using the Annexin V-FITC Apoptosis Detection Kit (Vazyme, A213 - 02). A253 cells were seeded in six-well plates (1 × 10^6 cells per well) and treated as described above. Cells were harvested, stained with Annexin V-FITC and propidium iodide, and analyzed using a BD LSRFortessa flow cytometer (USA). Data were processed using FlowJo software (n=3).

### 2.13 Molecular docking

Molecular docking was performed using Autodock Vina 1.2.2. The molecular structure of artesunate was obtained from the PubChem Compound Database, and protein structures for murine KEAP1 (PDB ID: 6zez) and human KEAP1 (PDB ID: 4ifn) were downloaded from the Protein Data Bank. Protein and ligand files were prepared by removing water molecules, adding polar hydrogen atoms, and converting files to PDBQT format. Docking grids were centered over the binding domains with dimensions of 30 Å × 30 Å × 30 Å and a grid spacing of 0.375 Å. Binding energies and interaction patterns were analyzed.

### 2.14 Western blotting

Protein lysates were prepared using RIPA buffer, and concentrations were determined using a BCA protein assay kit. Equal amounts of protein were resolved on 10% SDS-PAGE gels and transferred to PVDF membranes. Membranes were blocked with 5% BSA and incubated overnight at 4 °C with primary antibodies against AQP5 (1: 2000, Abclonal, A9927), GPX4 (1: 4000, Abclonal, A25009), TFRC (1: 1000, Abclonal, A25900), KEAP1 (1: 1000, Abclonal, A25951), NRF2 (1: 1000, Abclonal, A21176), and xCT (1: 1000, Abcam, ab307601). Horseradish peroxidase-conjugated secondary antibodies were applied, and signals were detected using enhanced chemiluminescence (ECL). Band intensities were quantified using ImageJ software (n=3).

### 2.15 Immunofluorescence staining

Tissue sections were deparaffinized, subjected to antigen retrieval, and blocked with 3% BSA for 30 minutes. Sections were incubated overnight at 4 °C with primary antibodies: anti-KEAP1 (1:200, Abclonal, cat. no. A25951), anti-xCT (1:300, Abcam, cat. no. ab307601), anti-GPX4 (1:200, Abclonal, cat. no. A25009), anti-TFRC (1:100, Abclonal, cat. no. A25900), and anti-NRF2 (1:100, Abclonal, cat. no. A0674). Secondary antibodies conjugated with Cy3 or Alexa Fluor 488 were incubated for 1 hour at room temperature. Nuclei were counterstained with DAPI, and images were captured using an Olympus FV3000 laser confocal scanning microscope; 20 images were randomly selected from different regions for analysis. For quantitative colocalization analysis of NRF2 subcellular distribution, refer to S2.

### 2.16 Statistical analysis

Data were analyzed using GraphPad Prism version 9 (GraphPad Software, San Diego, CA, USA). Results are presented as mean ± standard error of the mean (SEM). One-way ANOVA followed by Tukey’s *post hoc* test was used for multiple group comparisons. Statistical significance was defined as p < 0.05 (*p < 0.05, **p < 0.01, ***p < 0.001, ****p < 0.0001).

## 3 Result

### 3.1 Artesunate alleviates salivary gland damage in pSJD-like animals

**Figure 1:**
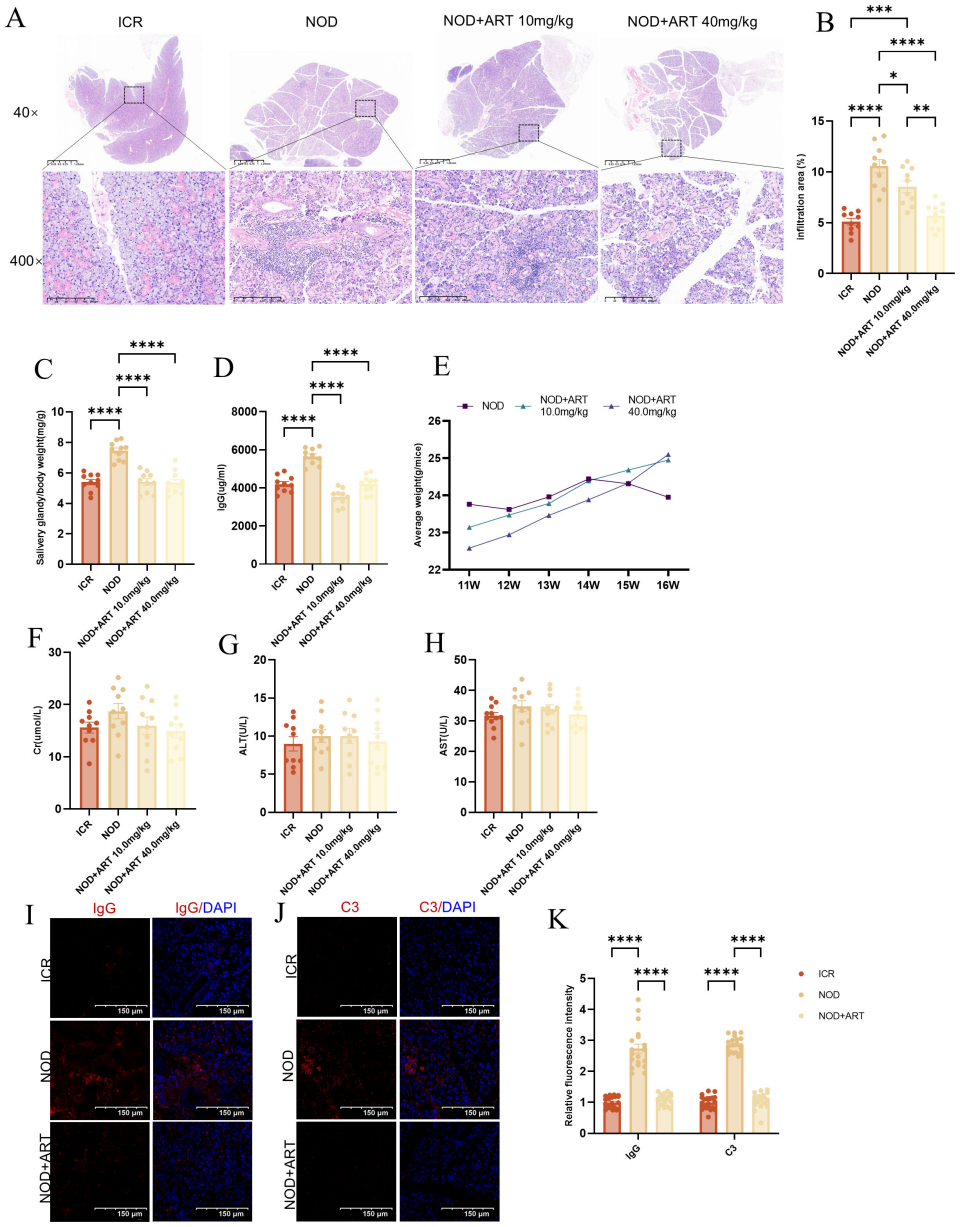
Artesunate Alleviates Salivary Gland Damage in pSJD-like animals. **(A)** H&E staining of submandibular glands (40×, scale bar=500μm; inset: 400×). **(B)** Quantification of inflammation area (%). **(C)** Salivary gland index (gland weight/body weight, n=10). **(D)** Plasma IgG levels (n=10). **(E)** Average body weight changes. **(F-H)** Plasma creatinine, ALT, and AST levels (n=10). **(I-K)** Confocal images of IgG and C3 deposition (scale bar=150µm) with semi-quantification (20 images from 3 mice/group, n=3). Data: mean ± SEM. *p<0.05, **p<0.01, ***p<0.001, ****p<0.0001; Ns, not significant.

ART treatment significantly reduced lymphocytic infiltration and improved salivary gland (SG) histopathology in NOD mice compared to untreated controls (**Figures 1A, B**). Quantification of inflammation areas confirmed these improvements (**Figure 1B**). Both ART doses (10 mg/kg and 40 mg/kg) significantly lowered SG indices compared to untreated NOD mice, though no significant differences were observed between the two ART doses (**Figure 1C**). ART-treated NOD mice exhibited significantly lower plasma IgG levels than vehicle-treated NOD mice, indicating suppression of immune overactivation (**Figure 1D**). ART-treated mice maintained stable growth trends despite being lighter than untreated groups before 15 weeks of age (22) (**Figure 1E**). No hepatorenal toxicity was observed, as plasma creatinine (Cr), ALT, and AST levels remained within normal ranges (**Figures 1F–H**). ART treatment markedly decreased IgG and complement C3 deposition in submandibular glands, reflecting inhibition of abnormal immune activation (**Figures 1I–K**). ART significantly reduces lymphocytic infiltration, SG indices, and autoantibody levels while maintaining safety profiles. These findings highlight ART as a promising therapeutic agent for SJD, with mechanisms involving immune modulation and functional restoration of SGs.

### 3.2 Artesunate rescues salivary gland epithelial cell viability and restores saliva secretion function

**Figure 2:**
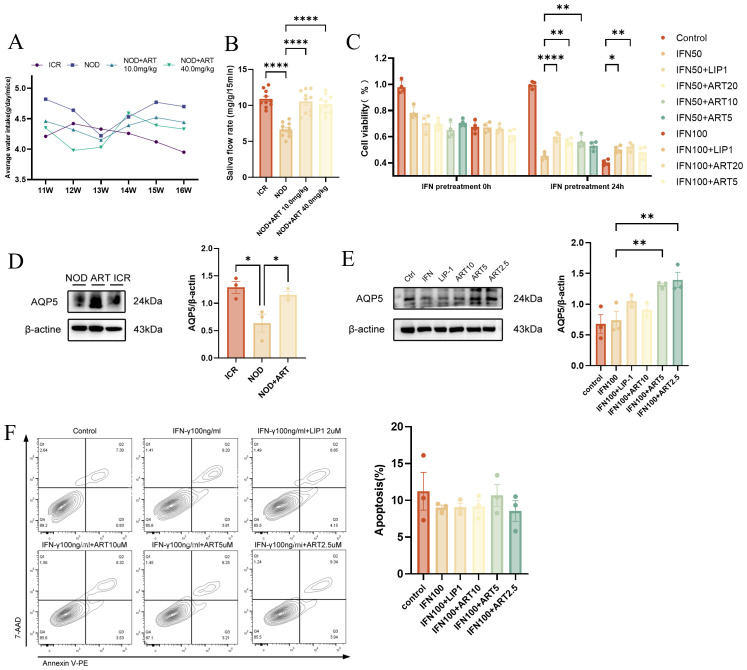
Artesunate Rescues Salivary Gland Epithelial Cell Viability and Restores Saliva Secretion Function. **(A)** Average water intake of ICR and NOD mice (*in vivo*). **(B)** Saliva flow rate (SFR, n=10) in mice (*in vivo*). **(C)** CCK - 8 assay for SGEC viability (human A253 cells, *in vitro*, n=4). **(D)** Western blot and quantification of AQP5 in mice (*in vivo*, n=3). **(E)** Western blot and quantification of AQP5 in SGECs (human A253 cells, *in vitro*, n=3). **(F)** Flow cytometry-based apoptosis assay (human A253 cells, *in vitro*, n=3). Data: mean ± SEM. *p<0.05, **p<0.01, ****p<0.0001; Ns, not significant.

ART-treated NOD mice showed a significant decrease in average water intake starting from week 14, maintaining stable levels between weeks 15 and 16 (**Figure 2A**). ART-treated mice exhibited significantly higher SFR compared to untreated NOD mice at 16 weeks of age (**Figure 2B**), indicating its potential therapeutic value. The CCK - 8 assay demonstrated that ART effectively rescued the decline in SGEC viability but did not prevent apoptosis (**Figure 2C**).ART treatment upregulated aquaporin 5 (AQP5) expression in salivary glands and SGECs, particularly at concentrations of 2.5 μM and 5 μM, enhancing saliva secretion capacity (**Figures 2D, E**).ART restored saliva secretion function without reducing apoptosis in SJD-related epithelial cells (**Figure 2F**). ART significantly increases saliva flow rate, indicating improved salivary secretion function. ART restores SGEC viability and enhances saliva secretion capacity by upregulating AQP5 expression. ART’s therapeutic effects are mediated through AQP5 upregulation rather than apoptosis reduction, revealing new mechanisms for its potential in treating SJD.

### 3.3 Single-cell transcriptomics dissects the gene of type I interferon-induced immune inflammation and ferroptosis in pSJD

**Figure 3:**
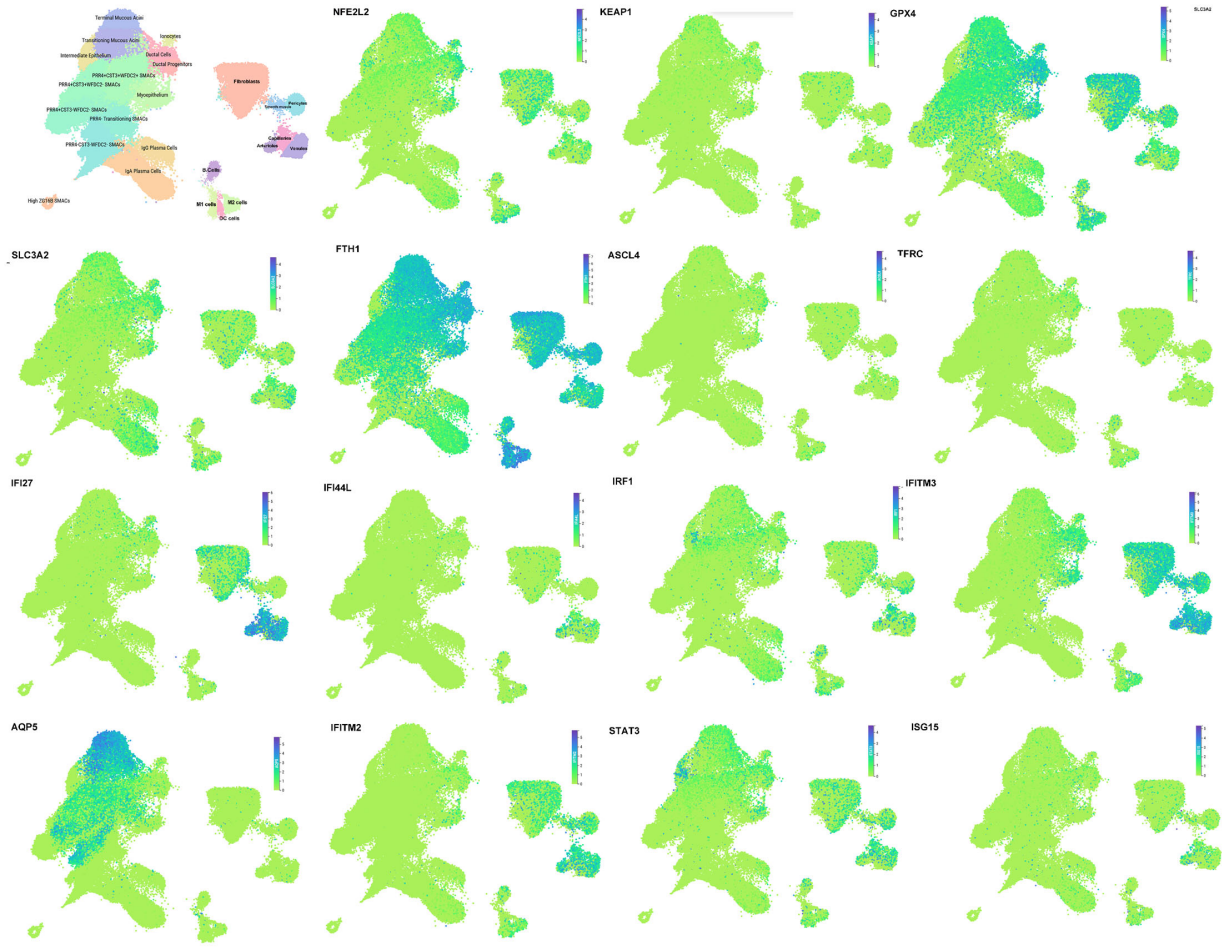
Single-cell transcriptomics dissects type I interferon-induced immune inflammation and ferroptosis-related genes in pSJD. Analysis of the GSE272409 dataset (7 SjD patients vs. 6 non-SjD sicca patients) identified 25 cell clusters, with parenchymal and immune cells showing enrichment of interferon-associated and ferroptosis-related genes. For cluster annotations, see **Supplementary Figure S3**.

By utilizing single-cell transcriptomic data from the GSE272409 dataset, which includes 7 patients with Sjögren’s disease (SjD) and 6 patients with non-SjD sicca, to define the cellular subpopulations in the salivary glands of patients with Sjögren’s Disease (SjD), we identified 25 distinct clusters through cell subpopulation clustering, followed by manual annotation based on the defined cell populations (**Figure 3**). These 25 cell types include Terminal Mucous Acini, Transitioning Mucous Acini lonocytes, Intermediate Epithelium, Ductal Cells, Ductal Progenitors, PRR4+CST3+WFDC2+ SMACs, PRR4+CST3+WFDC2-SMACs, PRR4+CST3-WFDC2-SMACs, PRR4-Transitioning SMACs, PRR4-CST3-WFDC2-SMACs, High ZG16B SMACs, Fibroblasts, Myoepithelium, Pericytes, Smooth Muscle, IgG Plasma Cells, IgA Plasma Cells, B Cells, M1 Macrophages, M2 Macrophages, Capillaries, Arterioles, Venules, and Dendritic Cells (DCs).The type I interferon gene subcluster (e.g., IFITM3, IFI44L, STAT3, ISG15, etc.) was significantly expressed in various immune cells, such as macrophages, B cells, and dendritic cells. Genes associated with ferroptosis (e.g., GPX4, NFE2L2, KEAP1, etc.) were prominently expressed in multiple parenchymal cells of the salivary gland, including myofibroblasts, fibroblasts, myoepithelial cells, ductal cells, and acinar cells.

### 3.4 Artesunate rescues ferroptosis in SGECs of pSJD-like animals

**Figure 4:**
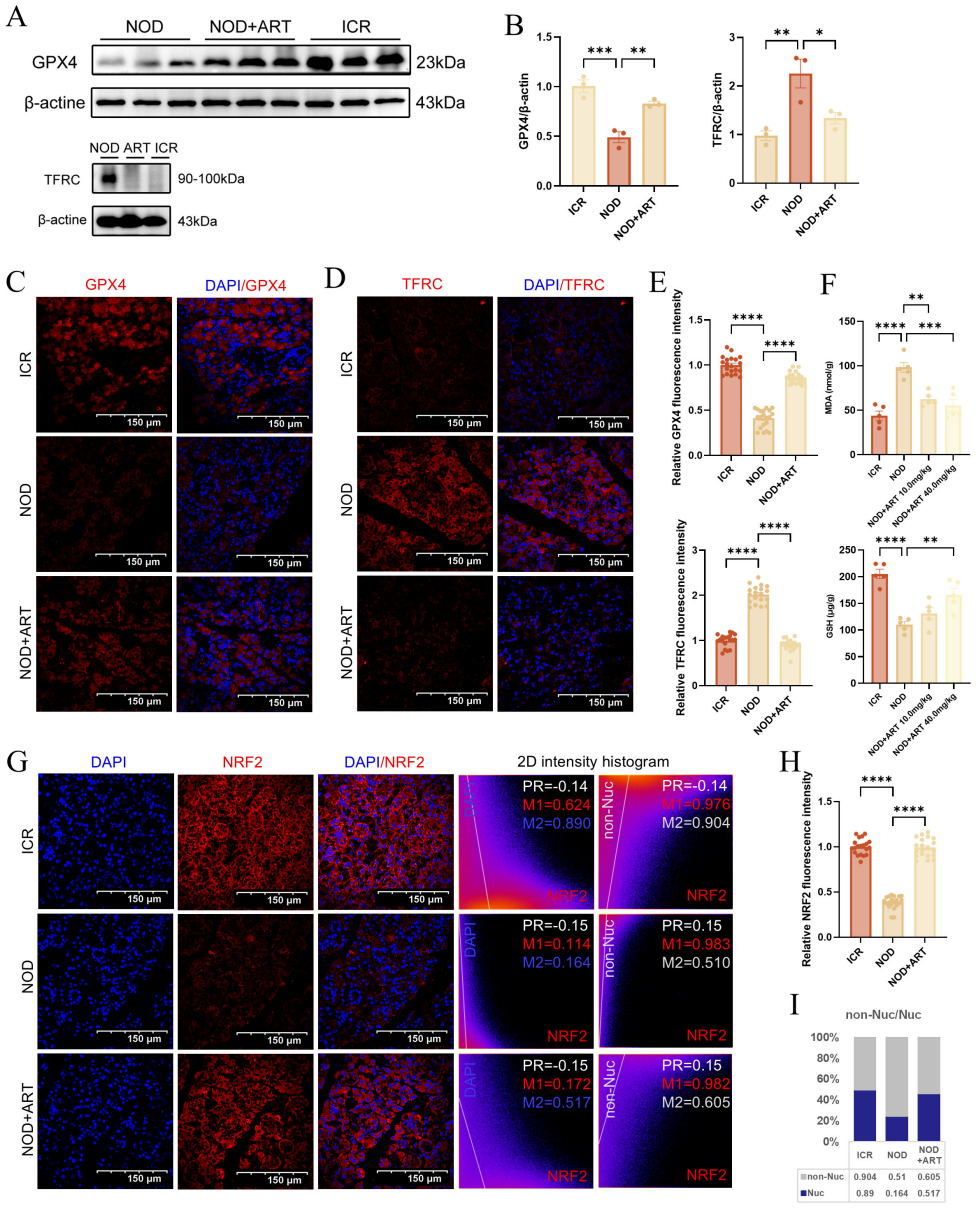
Artesunate Rescues Ferroptosis In Sgecs Of Psjd-Like Animals. **(A, B)** Western blot analysis and quantification of GPX4 and TFRC (n=3). **(C–E)** Confocal images of GPX4 and TFRC (scale bar=150μm) with semi-quantification of relative mean fluorescence intensity (MFI) (20 images from 3 mice/group, n=3). **(F)** The level of GSH and MDA in mice salivary gland tissues. **(G, H)** Confocal images of NRF2 (scale bar=150μm) with semi-quantification of relative mean fluorescence intensity (MFI) (20 images from 3 mice/group, n=3).In the 2D intensity histogram, PR represents Pearson’s Coefficient, M1 represents the red Manders’ Coefficients, and M2 represents the green Manders’ Coefficients. For the detailed analysis process of NRF2 subcellular distribution, see S2. Data: mean ± SEM. *p<0.05, **p<0.01, ***p<0.001, ****p<0.0001; Ns, not significant.

RNA-seq analysis reveals that artesunate regulates ferroptosis-related pathways including lipid peroxidation, reactive oxygen species generation, and iron ion transport (**Supplementary Figure S1**), indicating the critical role of ferroptosis in the pathogenesis of Sjögren’s Disease. In pSJD model mice, GPX4 expression was significantly downregulated, while TFRC expression was markedly upregulated, indicating ferroptotic changes in salivary gland epithelial cells (SGECs). ART treatment reversed these changes, significantly upregulating GPX4 and suppressing TFRC expression, suggesting its protective role against ferroptosis (**Figures 4A, B**). Immunofluorescence staining confirmed that ART restored GPX4 and TFRC levels to near-normal levels, comparable to those in ICR control mice (**Figures 4C–E**). Consistent with the ferroptotic phenotype, pSJD model mice exhibited significantly elevated malondialdehyde (MDA) levels and reduced glutathione (GSH) levels in salivary gland tissues compared to ICR controls; ART treatment effectively normalized these oxidative stress markers, restoring them to levels close to those in healthy mice (**Figure 4F**). NRF2, a key regulator of ferroptosis, was significantly downregulated in the pSJD group. Manders’ Coefficients analysis revealed that the M2 ratio (non-nuclear to nuclear localization) of NRF2 in the NOD group was significantly lower than that in the ICR group (3.110 vs. healthy baseline), indicating reduced nuclear translocation of NRF2 and increased extranuclear retention in pSJD. Notably, ART treatment restored this ratio to 1.170, approaching healthy levels (**Figures 4G–I**). These findings indicate that ART mitigates ferroptosis in SGECs by modulating GPX4 and TFRC expression, regulating oxidative stress markers (GSH and MDA), and promoting NRF2 expression and nuclear translocation, thereby inhibiting ferroptosis through the NRF2-GPX4 axis.

### 3.5 Artesunate inhibits IFN-γ-induced ferroptosis in SGECs

**Figure 5:**
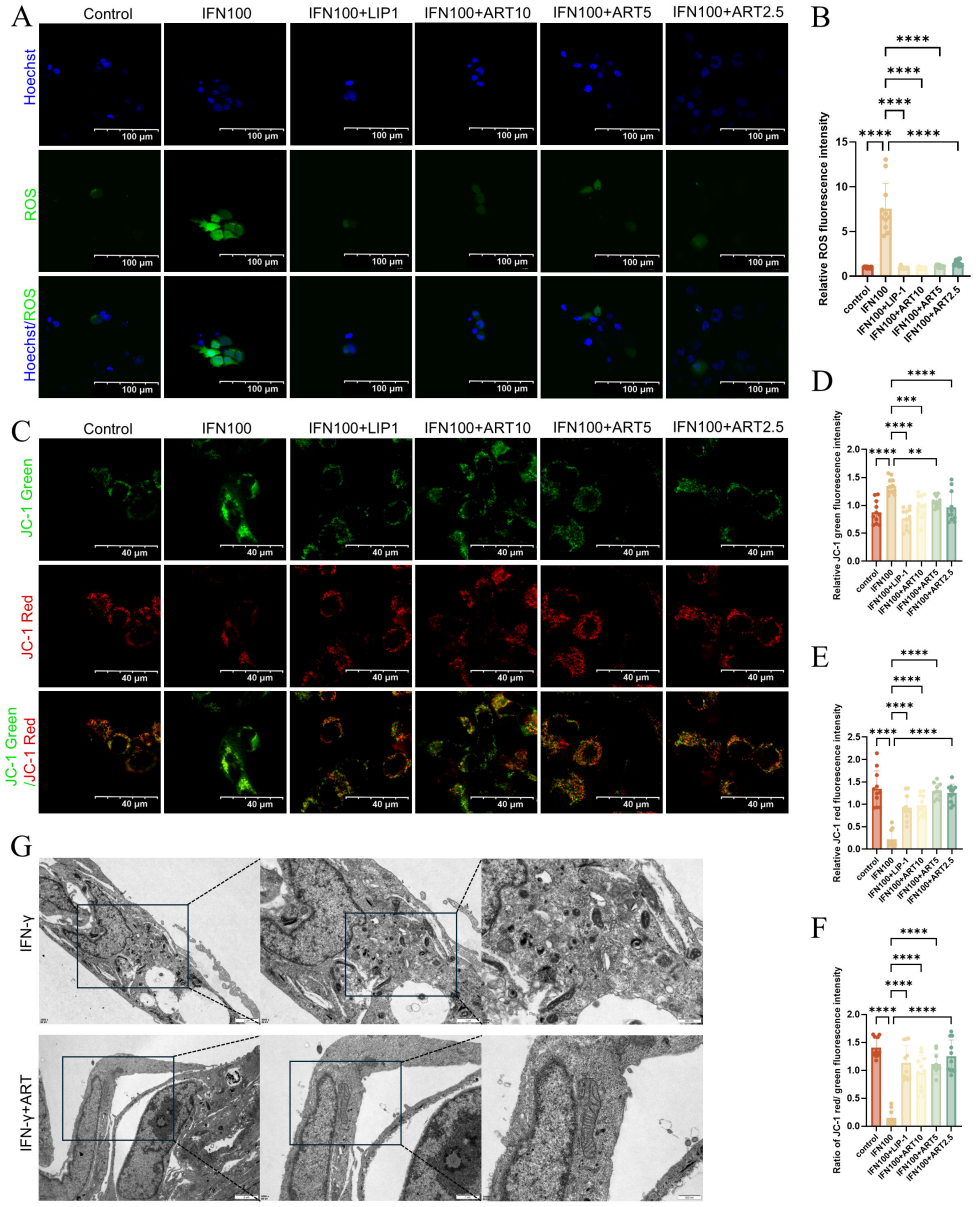
Artesunate Inhibits IFN-γ-Induced Ferroptosis in SGECs. **(A, B)** ROS levels (scale bar=100μm) with semi-quantification of relative mean fluorescence intensity (MFI) (11 images from 3 samples/group, n=3). **(C-F)** JC - 1 Red/Green fluorescence (scale bar=40μm) with semi-quantification of relative mean fluorescence intensity (MFI) (11 images from 3 samples/group, n=3). **(G)** Representative TEM images of SGECs of mitochondrial morphology. Data: mean ± SEM. **p<0.01, ***p<0.001, ****p<0.0001; Ns, not significant.

ART significantly reduced ROS levels elevated by IFN-γ, with 10 μM ART achieving effects comparable to Liproxstatin-1, a known ferroptosis inhibitor (**Figures 5A, B**).This indicates that ART effectively mitigates oxidative stress induced by IFN-γ. Using JC - 1 dye, ART restored the weakened MMP caused by IFN-γ exposure. At 5 μM, ART demonstrated slightly better MMP restoration compared to Liproxstatin-1, as evidenced by increased red mitochondrial fluorescence (**Figures 5C–F**).Transmission electron microscopy (TEM) revealed severe mitochondrial damage in IFN-γ-treated SGECs, including contraction, membrane rupture, and cristae degradation. ART treatment at 5 μM significantly improved mitochondrial morphology, restoring cristae structure and reducing matrix darkening (**Figure 5G**).This study highlights ART’s dual role in mitigating oxidative stress and preserving mitochondrial integrity, providing mechanistic insights into its anti-ferroptotic properties.

### 3.6 Artesunate inhibits IFN-γ induced SGECs ferroptosis via the KEAP1-NRF2-xCT axis

**Figure 6:**
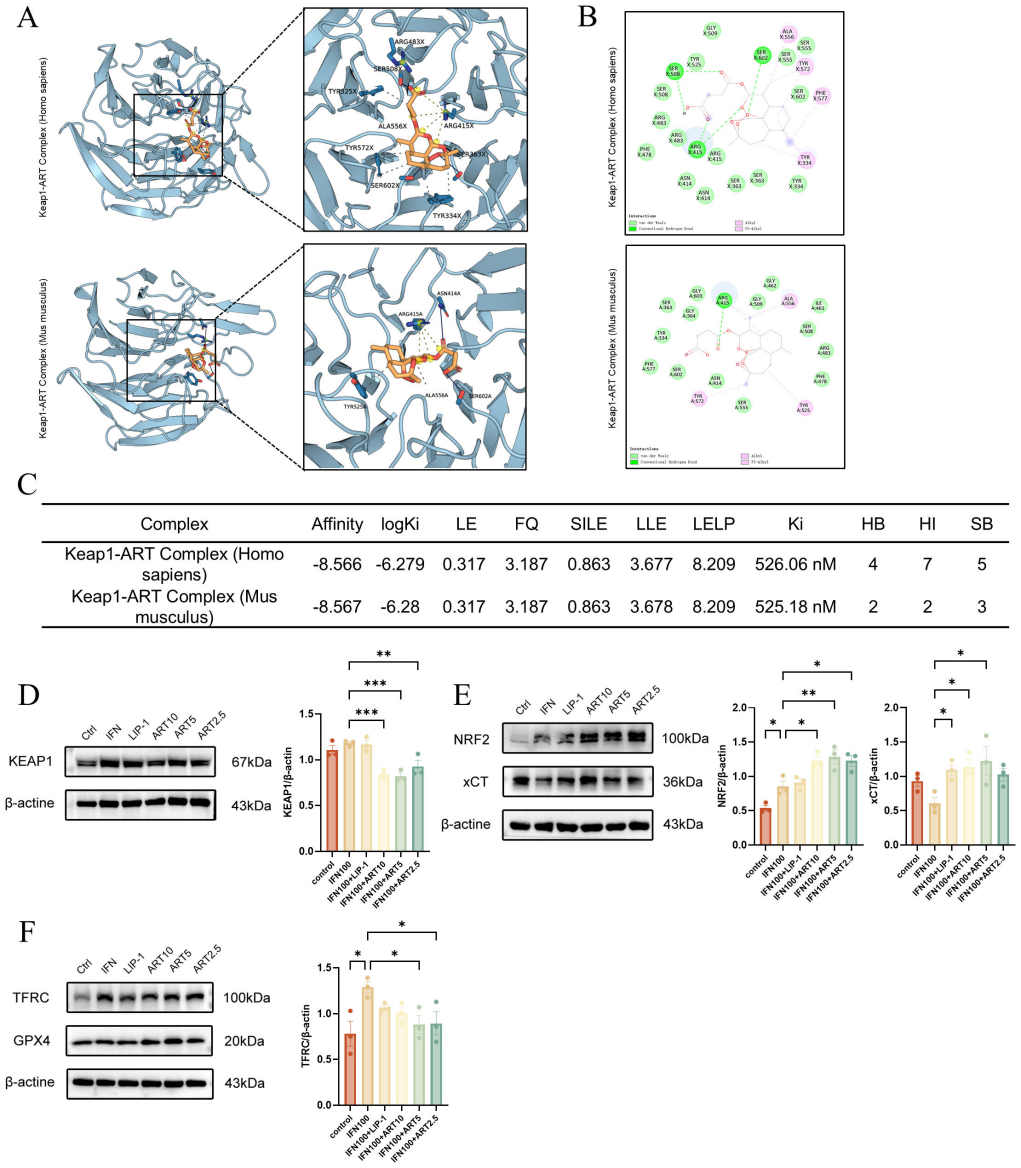
Artesunate inhibits IFN-γ induced SGEC ferroptosis via the KEAP1-NRF2-xCT axis. **(A, B)** Molecular docking analysis of artesunate with human/mouse KEAP1: **(A)** Left: best docking conformation of the KEAP1-artesunate complex; Right: 3D molecular interactions identified by PLIP (blue line: hydrogen bond; gray dashed line: hydrophobic interaction; yellow line: salt bridge); **(B)** 2D interaction diagram generated by Discovery Studio. **(C)** Key docking metrics, including binding affinity (ΔG, kcal/mol), logarithmic inhibition constant (logKi, M), efficiency indices (LE, SILE, LLE, LELP), and interaction types (HB: hydrogen bonds, HI: hydrophobic interactions, SB: salt bridges; calculated via AutoDock Vina). **(D-F)** Western blot and quantification of ferroptosis-related proteins (TFRC, GPX4) and KEAP1-NRF2-xCT axis components (KEAP1, NRF2, xCT) (n=3). Data are presented as mean ± SEM. *p<0.05, **p<0.01, ***p<0.001; Ns, not significant.

ART bound human/mouse Keap1 with near-identical affinity (ΔG: -8.57/-8.58 kcal/mol), supported by nanomolar Ki (526/525 nM) and conserved ligand efficiency (LE: 0.317 kcal/mol·atom, 28 non-H atoms). Structural analyses revealed 4 H-bonds (SER - 508/ARG-415) and 7 hydrophobic interactions in human Keap1, anchoring ART in the Kelch domain critical for NRF2 ubiquitination (**Figures 6A - C**) (27). ART significantly downregulated Keap1 expression while upregulating NRF2 and xCT protein levels in IFN-γ-induced salivary gland epithelial cells (SGECs). These effects were dose-dependent and superior to those of Liproxstatin-1 (**Figures 6D, E**).ART also decreased the ferroptosis marker TFRC and increased GPX4 expression, with slightly better outcomes than Liproxstatin-1 (**Figure 6F**). ART inhibits Keap1-mediated ubiquitination and degradation of NRF2 in the cytoplasm, thereby activating NRF2-regulated antioxidant gene expression. This mechanism explains ART’s ability to rescue SGECs from ferroptosis. These findings provide mechanistic insights into ART’s protective effects on SGECs, highlighting its therapeutic potential in mitigating ferroptosis-related damage in salivary glands.

### 3.7 Artesunate rescues SGECs of pSJD-like animal from ferroptosis via the KEAP1-NRF2-xCT pathway

**Figure 7:**
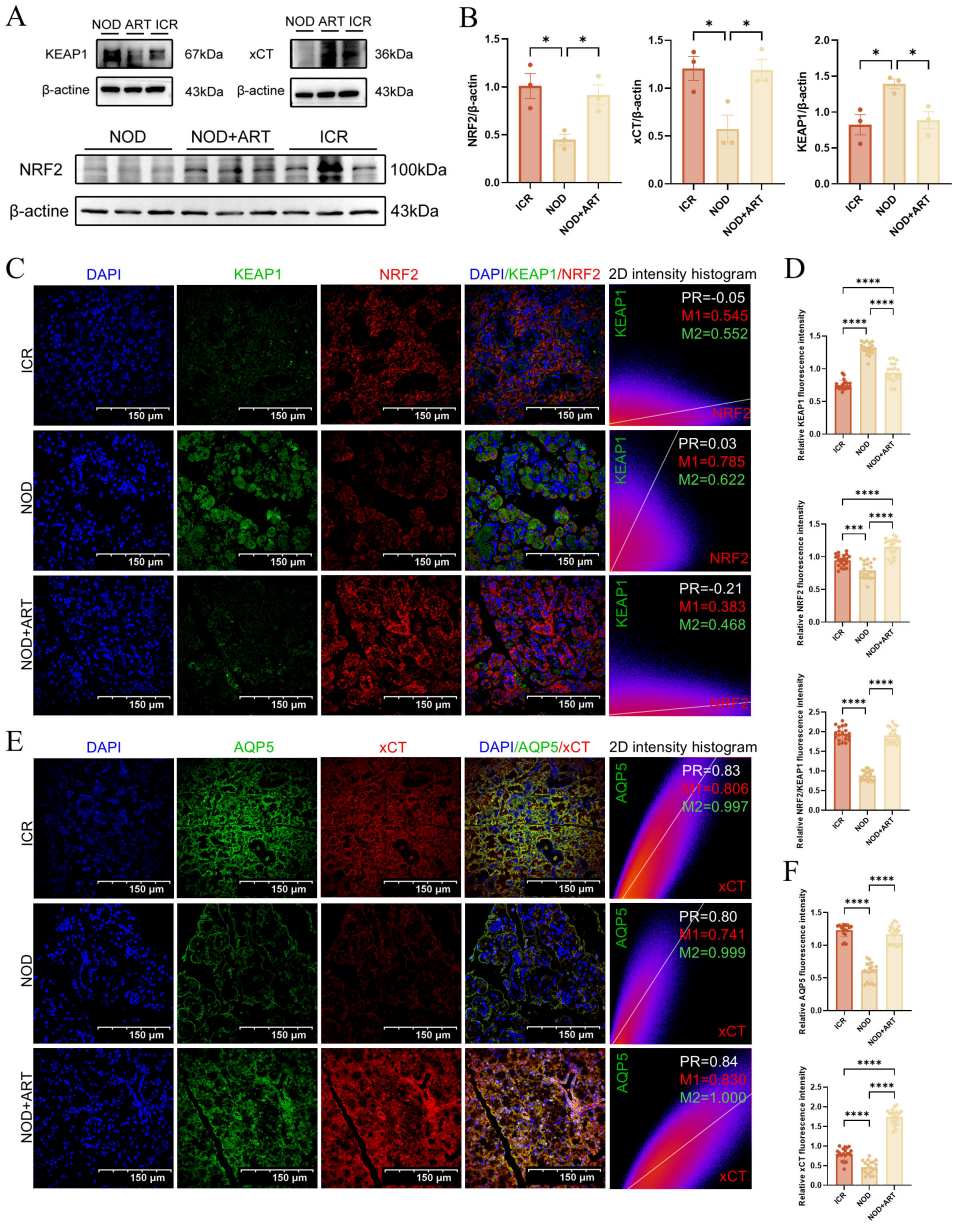
Artesunate Rescues SGECs of pSJD-like animals from Ferroptosis via the KEAP1-NRF2-xCT Pathway (3). **(A, B)** Western blot and quantification of KEAP1, NRF2, and xCT (n=3). **(C, D)** Confocal images of KEAP1-NRF2 colocalization (scale bar=150μm) with Manders’ coefficients (20 images from 3 mice/group, n=3). **(E, F)** Confocal images of xCT-AQP5 colocalization (scale bar=150μm) with Manders’ coefficients (20 images from 3 mice/group, n=3). In the 2D intensity histogram, PR represents Pearson’s Coefficient, M1 represents the red Manders’ Coefficients, and M2 represents the green Manders’ Coefficients. Data: mean ± SEM. *p<0.05, ****p<0.0001; Ns, not significant.

Western Blotting demonstrated that ART significantly inhibited KEAP1 expression while upregulating NRF2 and xCT in the submandibular glands of pSJD model mice, restoring levels to those observed in ICR control mice (**Figures 7A, B**).Immunofluorescence confirmed that ART reversed the downregulation of NRF2 and upregulation of KEAP1 in pSJD mice. Colocalization analysis using Manders’ Coefficients revealed strong KEAP1-NRF2 binding in pSJD mice (M1 = 0.785), which was reduced by ART treatment (**Figures 7C, D**).Immunofluorescence analysis showed that ART significantly rescued the downregulation of xCT and AQP5 in pSJD models. Strong colocalization between xCT and AQP5, as measured by Pearson’s Correlation Coefficient and Manders’ Coefficients, indicated ART’s role in mitigating ferroptosis and restoring salivary secretion capacity (**Figures 7E, F**).ART inhibits KEAP1-mediated ubiquitination and degradation of NRF2, activating NRF2-regulated antioxidant gene expression and rescuing SGECs from ferroptosis. These findings elucidate ART’s therapeutic mechanisms in pSJD. This study provides mechanistic insights into ART’s protective effects on SGECs, offering new directions for future research and therapeutic development in pSJD.

## 4 Discussion

Sjögren’s Disease (SjD) is an autoimmune disease primarily characterized by dysfunction of the salivary and lacrimal glands, significantly impacting patients’ quality of life (28). Despite recent advances in understanding the pathophysiology of SJD, there remains a lack of FDA-approved drugs capable of effectively treating this condition (29). Consequently, the development of novel therapeutic strategies has become a focal point of current research. This study focuses on Artesunate (ART), a compound derived from the traditional Chinese medicinal herb Artemisia annua, to explore its therapeutic potential and underlying mechanisms in a non-obese diabetic (NOD) mouse model of SJD. Our findings demonstrate that ART protects salivary gland epithelial cells (SGECs) and restores their secretory function by modulating the KEAP1-NRF2-xCT pathway to inhibit ferroptosis. The implications of these findings are discussed below from multiple perspectives.

Our results indicate that ART significantly alleviates salivary gland damage in NOD mice and reduces serum IgG levels, suggesting its ability to suppress excessive immune responses. Furthermore, ART decreases the deposition of IgG and complement C3 in salivary gland tissues, further confirming its anti-inflammatory effects. These data collectively demonstrate that ART not only mitigates inflammation associated with SJD but also reduces immune-mediated tissue destruction. Notably, ART treatment did not induce significant hepatorenal toxicity, providing a safety assurance for its potential use as a therapeutic agent. In terms of functional assessment, ART significantly improved the saliva flow rate (SFR) in NOD mice and restored the viability of SGECs. This improvement is closely associated with the upregulation of AQP5 expression, a key water channel protein essential for maintaining salivary secretion. However, ART did not significantly reduce apoptosis in SGECs, indicating that its protective effects are primarily mediated through alternative mechanisms, such as the inhibition of ferroptosis. These findings lay the groundwork for understanding the multi-target therapeutic actions of ART in SJD.

Using single-cell RNA sequencing (scRNA-seq), we reanalyzed data from Nayar et al. (26), to characterize the expression profiles of type I interferon and ferroptosis-related gene sets in both parenchymal and immune cells of the salivary glands. We identified several pathogenic cell subsets in the salivary glands of pSJD patients, including endothelial cells, macrophages, dendritic cells, CD4+ T cells, and CD8+ T cells. These cell types play critical roles in SJD progression by promoting inflammatory cytokine release and inducing tissue damage (26). Specifically, we found that type I interferon gene clusters (e.g., IFITM3, IFI44L, STAT3, ISG15) were significantly expressed in various immune cells, such as macrophages, B cells, and dendritic cells. Meanwhile, ferroptosis-related genes (e.g., GPX4, NFE2L2, KEAP1) were prominently expressed in multiple parenchymal cells of the salivary glands, including myofibroblasts, fibroblasts, myoepithelial cells, ductal cells, and acinar cells. Dysregulation of type I interferons (IFNs) is a hallmark of SJD, with abnormal activation of the type I IFN pathway leading to chronic inflammation and fibrosis of the salivary glands (16, 30). Our previous study (19) revealed that ART may achieve overall control of SJD by inhibiting the type I IFN signaling pathway and blocking downstream inflammatory cascades.

Ferroptosis, a form of regulated cell death driven by lipid peroxidation, plays a pivotal role in the pathogenesis of Sjögren’s syndrome (SJD). Interferon-γ induces ferroptosis in salivary gland epithelial cells in Sjögren’s syndrome through JAK/STAT1-mediated inhibition of system Xc (23). Downregulation of GPX4 in salivary gland epithelial cells leads to salivary secretion dysfunction in Sjögren’s syndrome via the lipid ROS/pSTAT4/AQP5 axis (11). Moreover, studies have shown that plasma exosomes from patients with primary Sjögren’s syndrome—represented by ceruloplasmin (CP) and transferrin (TF)—contain epithelial cell-derived proteins involved in ferroptosis (31). Research has demonstrated (32) that autologous serum from patients with Sjögren’s syndrome alleviates hypertonicity-induced corneal epithelial ferroptosis by inhibiting the Xc/GPX4 pathway. In the field of stem cell therapy for Sjögren’s syndrome, hypoxic ADSC-derived exosomes attenuate skin damage caused by primary Sjögren’s syndrome through GLRX2 delivery and ferroptosis suppression (33). Exosomes derived from human exfoliated deciduous teeth (SHED-exos) treatment improved salivary flow rates in NOD mice, accompanied by reduced levels of cleaved caspase-3 and decreased numbers of apoptotic cells in submandibular glands (SMGs). SHED-exos suppress ferroptosis, necrosis, and expression of oxidative stress markers in damaged salivary glands in Sjögren’s syndrome (34). Melatonin inhibits ferroptosis in salivary gland epithelial cells in primary Sjögren’s syndrome via the NRF2/HO-1/GPX4 signaling pathway, thereby attenuating ferroptosis-triggered NF-κB activation (35). In the realm of natural compounds, recent studies have revealed that apigenin modulates Sjögren’s syndrome-induced ferroptosis in salivary gland epithelial cells through the ERα signaling-mediated ATF3/SLC7A11 axis (14). Genistein exerts protective and anti-inflammatory effects on salivary glands in Sjögren’s syndrome by inhibiting Xist/ACSL4-mediated ferroptosis following binding to estrogen receptor-α (36).

In this study, we found that ART significantly downregulated TFRC (transferrin receptor) expression while upregulating GPX4 (glutathione peroxidase 4) and NRF2 (nuclear factor E2-related factor 2) expression, thereby suppressing ferroptosis in SGECs. Specifically, ART exerts its effects through the following mechanisms (1): Inhibition of TFRC Expression: TFRC facilitates intracellular iron uptake, and its overexpression increases cellular susceptibility to ferroptosis (37). ART significantly reduced TFRC levels, thereby decreasing intracellular iron overload (2). Enhancement of Antioxidant Defense: GPX4 is a key antioxidant enzyme that protects cells from ferroptosis by scavenging harmful lipid peroxides (14). ART markedly increased GPX4 expression, enhancing cellular antioxidant capacity (3). Activation of the NRF2 Signaling Pathway: NRF2 is a central transcription factor regulating antioxidant gene expression. Under normal conditions, NRF2 is rapidly degraded upon binding to its negative regulator KEAP1 (38). However, ART treatment weakened the interaction between KEAP1 and NRF2, allowing NRF2 to translocate into the nucleus and activate downstream antioxidant genes. Molecular docking experiments further confirmed the high-affinity binding of ART to KEAP1, suggesting that ART may directly target the KEAP1-NRF2 axis to inhibit ferroptosis. This finding provides a theoretical basis for developing new therapies targeting ferroptosis (4). Mitochondrial Protection and Comparative Efficacy: Mitochondrial dysfunction, a hallmark of ferroptosis (39), was reversed by ART in IFN-γ-treated SGECs. JC - 1 staining and transmission electron microscopy revealed restored mitochondrial membrane potential and structural integrity (e.g., cristae preservation). Compared to the ferroptosis inhibitor Liproxstatin-1 (40), ART exhibited superior efficacy in modulating NRF2 and xCT expression. xCT, a component of the cystine/glutamate antiporter, facilitates glutathione synthesis to counteract oxidative stress. ART-driven xCT upregulation highlights its multi-targeted antioxidant synergy.

## 5 Conclusion

This study systematically elucidates ART’s mechanism in SJD: ferroptosis inhibition via KEAP1-NRF2-xCT pathway modulation, leading to SGEC protection and salivary function restoration. These findings advance SJD pathology understanding and offer novel therapeutic avenues. As a naturally derived small molecule, ART holds promise as a groundbreaking candidate for SJD treatment.

Strengths, Limitations, and Future Directions: While our findings are primarily derived from animal models and *in vitro* studies, they provide a robust foundation for ART’s therapeutic potential in SJD. ART’s established safety in malaria and inflammatory diseases and its multi-targeted action—combining anti-inflammatory and cytoprotective effects—address limitations of conventional immunosuppressants. However, clinical translation requires large-scale human trials to assess long-term efficacy, safety, and optimized dosing regimens. Future studies should explore ART-based combination therapies to enhance outcomes.

## Data Availability

The data presented in the study are deposited in the NCBI GEO repository (accession number GSE272409) and NCBI BioProject (accession number PRJNA1314776).
