# Supplementary material for: Artemisinin derivatives modulate KEAP1-NRF2-xCT pathway to alleviate Sjögren’s disease: insights from scRNA-seq and systems biology
Source: Front Immunol. 2025 Sep 9;16:1626230. doi: 10.3389/fimmu.2025.1626230 (PMC12418045; doi:10.3389/fimmu.2025.1626230)

S1. . Transcriptomics Reveals Artesunate Regulates Ferroptosis and Oxidative Stress to Restore Salivary Gland Function

RNA-seq analysis identified 311 DEGs in salivary glands (SGs) from ART-treated NOD mice compared to vehicle controls, with 130 downregulated and 81 upregulated genes (S1. A).Heatmaps highlighted the top 30 most significantly altered genes, visually demonstrating ART's impact on SG gene expression patterns (S1. B).GO enrichment analysis revealed significant modulation of ferroptosis-related pathways across molecular function (MF), cellular component (CC), and biological process (BP) categories (S1. C, D).Upregulated genes were enriched in pathways related to lipid peroxidation, reactive oxygen species (ROS) generation, and iron ion transport—key features of ferroptosis.Downregulated genes were primarily associated with inflammatory and immune responses, consistent with ART's immunomodulatory effects.KEGG and Reactome pathway analyses confirmed ART's regulation of ferroptosis and oxidative stress-related pathways.ART modulated critical molecular events, such as activating lipid metabolism and inhibiting inflammatory signaling.ART restores SG function by targeting ferroptosis and oxidative stress pathways, providing a mechanistic basis for its therapeutic effects in Sjögren's Disease (SjD).These findings support ART as a promising candidate for developing novel SJD therapies (S1. E-G).This study provides robust evidence supporting ART's potential as a treatment for SJD, emphasizing its role in modulating ferroptosis and oxidative stress.

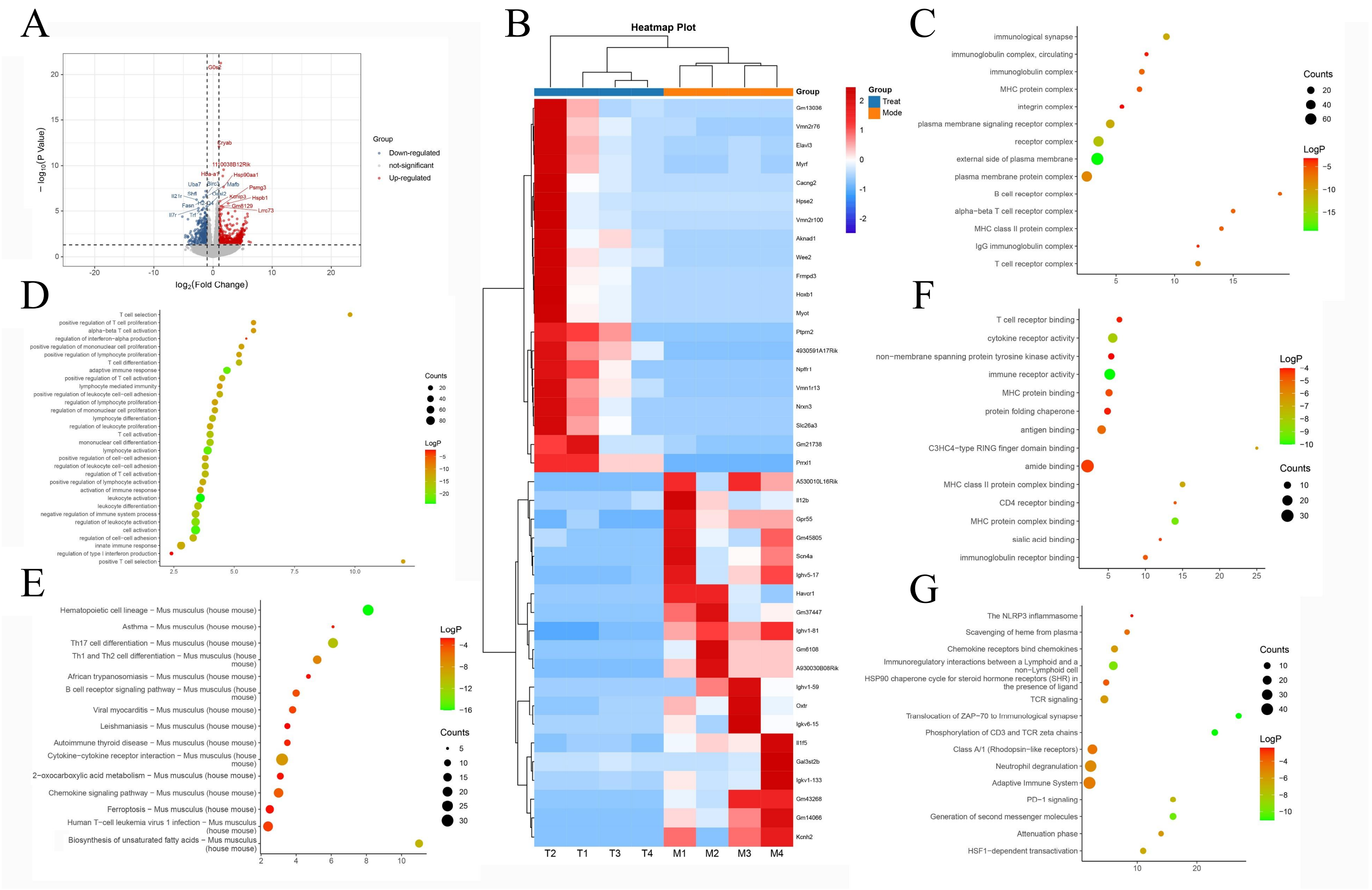

S1. . Transcriptomics Reveals Artesunate Regulates Ferroptosis and Oxidative Stress to Restore Salivary Gland Function. (A) Volcano plot of differentially expressed genes (DEGs). (B) Heatmap of top DEGs in ART-treated (n=4) vs. vehicle-treated NOD mice (n=4; |log$_2$Fold change|≥2, p<0.05); transcriptome samples were randomly selected from 10 mice per group. (C-E) GO enrichment analysis (molecular function, cellular component, biological process). (F-G) KEGG and Reactome pathway analysis of DEGs.

S2. Quantitative Colocalization Analysis of NRF2 Subcellular Distribution

Quantitative colocalization analysis was performed using Fiji (a distribution of ImageJ, version 20250805) integrated with the Coloc 2 plugin (https://imagej.net/Coloc_2), a validated tool for quantitative colocalization assessment that incorporates statistical and visualization algorithms.

Multichannel fluorescent images were split into two distinct channels: the red channel (fluorescently labeled NRF2) and the blue channel (DAPI, a nuclear marker). Nuclear regions of interest (ROIs) were demarcated based on the original DAPI channel, where high-intensity pixels corresponded to nuclear regions. For cytoplasmic ROIs, the DAPI channel was processed via the "Invert" function in Fiji, which converted high-intensity nuclear regions to low-intensity background and low-intensity cytoplasmic regions to high intensity, thereby indirectly defining cytoplasmic areas.

In Coloc 2, channel assignments were specified as follows: for nuclear analysis, Channel 1 (NRF2, red) and Channel 2 (original DAPI, blue); for cytoplasmic analysis, Channel 1 (NRF2, red) and Channel 2 (inverted DAPI, blue). ROIs were designated as "Whole image," with the inverted DAPI channel inherently restricting cytoplasmic analysis to the cytoplasmic compartment. Analyses utilized default parameters for threshold regression (Bisection) and point spread function (PSF = 3.0), encompassing Spearman’s rank correlation coefficient (PR) to quantify monotonic pixel intensity correlations (robust to outliers), Manders’ correlation coefficients (M_1 for the NRF2 channel, M_2 for the DAPI channel) to determine the proportion of colocalized pixels in each channel, and 2D intensity histograms to visualize bivariate pixel intensity distributions and characterize colocalization patterns (e.g., clustering, dispersion).

A

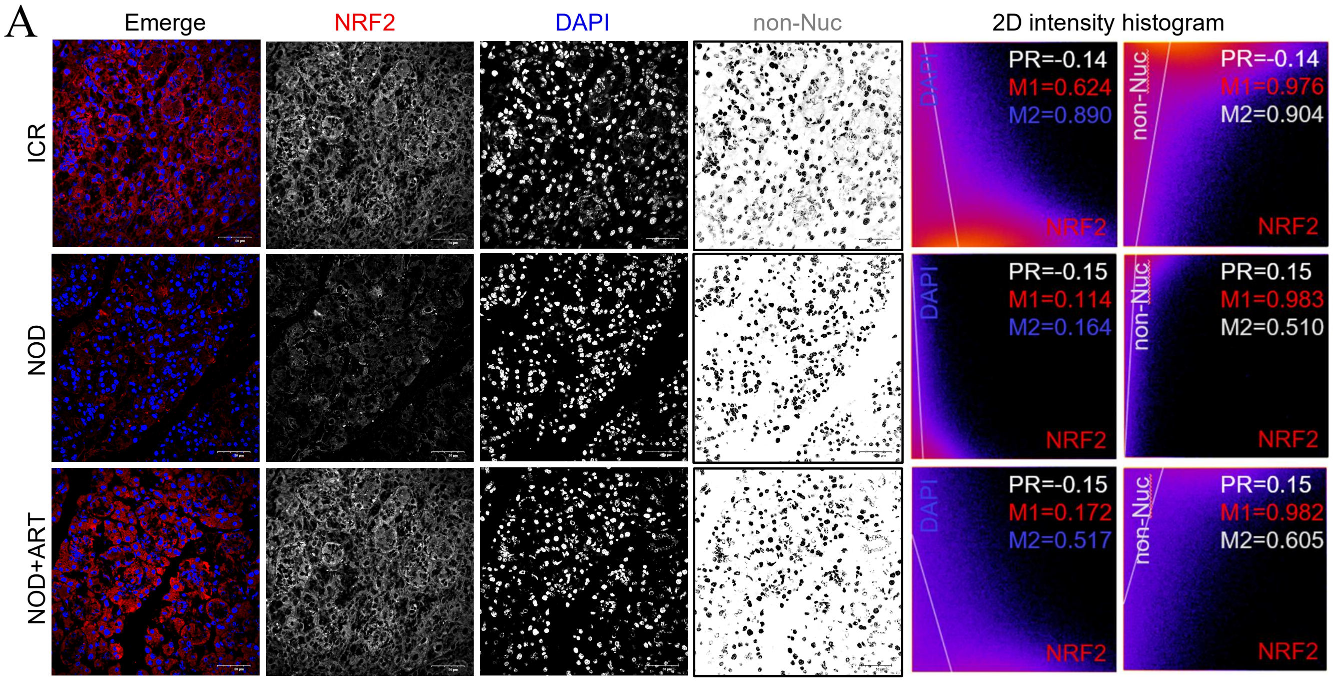

B

$$\mathrm{PCC} = \frac{\sum_i (R_i - \bar{R}) \times (G_i - \bar{G})}{\sqrt{\sum_i (R_i - \bar{R})^2 \times \sum_i (G_i - \bar{G})^2}}$$

C

$$M_1 = \frac{\sum_i R_{i,\mathrm{colocal}}}{\sum_i R_i} \qquad M_2 = \frac{\sum_i G_{i,\mathrm{colocal}}}{\sum_i G_i}$$

S2. Quantitative Colocalization Analysis of NRF2 Subcellular Distribution. (A) Confocal images of NRF2, red single channel, blue single channel and blue inverted single channel (scale bar=50μm). 2D intensity histogram: PR (Pearson's Coefficient), M1 (red Manders' Coefficient), M2 (green Manders' Coefficient). (B) Pearson's Correlation Coefficient (PCC, Rr) quantifies linear correlation between red and green channel pixel intensities (Ri/Gi: red/green intensity of pixel i; R/G: mean red/green intensity). (C) Manders’ Coefficients (MCC): M1 = proportion of red channel signal colocalizing with green; M2 = proportion of green channel signal colocalizing with red. M1 and M2 are typically distinct, quantifying colocalization in the region of interest.

S3. Annotations are used to create 25 clusters for 3.3 Single - cell transcriptomics.

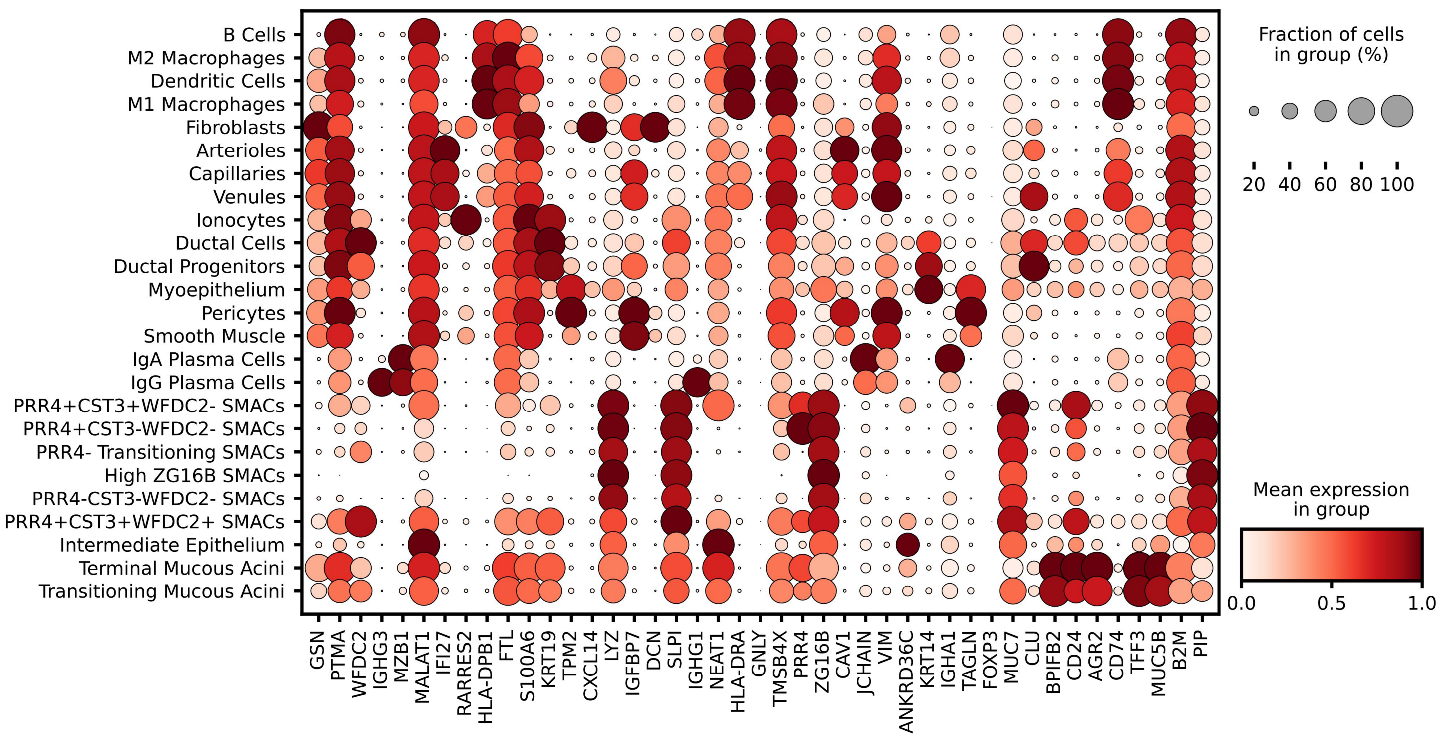

Supplement: Supplementary file 1 [file DataSheet1.pdf]
